# Seromucinous Hamartoma of the Lateral Nasal Wall with Infiltration of the Orbit: A Rare Case Report and Review of the Literature

**DOI:** 10.1155/2023/1923015

**Published:** 2023-08-12

**Authors:** Lentiona Basiari, Maria Michali, Ioannis Komnos, Georgios Tsirves, Victoria Tsoumani, Ioannis Kastanioudakis

**Affiliations:** Department of Otorhinolaryngology-Head and Neck Surgery, University Hospital of Ioannina, Ioannina, Greece

## Abstract

Seromucinous hamartoma is a rare benign glandular proliferation arising from the respiratory epithelium of the sinonasal tract and nasopharynx. It was described for the first time in 1974 by Baillie and Batsakis. Since then, few cases have been reported in the literature with most of them occurring in the posterior nasal septum. We report the case of a 52-year-old woman that presented to our department with left periorbital edema, pain, and dacryorrhea due to seromucinous hamartoma arising from the left inferior turbinate and extending through the lateral nasal wall into the maxilla, the nasolacrimal duct, and the orbit. Endoscopic medial maxillectomy and endoscopic transnasal orbital tumor resection were performed. The patient remains symptom-free for 16 months, till her most recent follow-up. Seromucinous hamartoma of the nasal cavity is an exceedingly rare diagnosis, especially in the lateral nasal wall. It should be included in the differential diagnosis of nasal tumors. According to the literature review, this is the first case report of seromucinous hamartoma with orbit infiltration. Endonasal endoscopic resection is the treatment of choice.

## 1. Introduction

Hamartomas of the sinonasal region can have an epithelial, mesenchymal, or mixed origin, and they include nasal chondromesenchymal hamartoma, respiratory epithelial adenomatoid hamartoma (REAH), seromucinous hamartoma (SH), and mixed chondroosseous respiratory epithelial hamartoma [1–3]. Although each of these has characteristic features, occasionally lesions might show overlapping features of multiple types [4]. They are benign neoplasms and recurrence is unusual. SH is the newest among these entities and was first described in 1974 by Baillie and Batsakis [5]. It is a rare benign glandular proliferation of the sinonasal tract and rarely of the nasopharynx, also known as serous hamartoma, glandular hamartoma, and microglandular adenosis. SH involves lesions originating from the epithelium, and most of them occur on the posterior nasal septum [6–9]. Most patients are middle or advanced age, with approximately the same incidence between sexes [6, 10]. Patients usually present with a long-standing history of nasal obstruction, mucopurulent secretion, and/or epistaxis [11]. In this article, we present the rare case of a seromucinous hamartoma of the lateral nasal wall with invasion of the orbit. Based on our research, this is the first case report of sinonasal SH that involves the orbit.

## 2. Case Report

**Figure 1:**
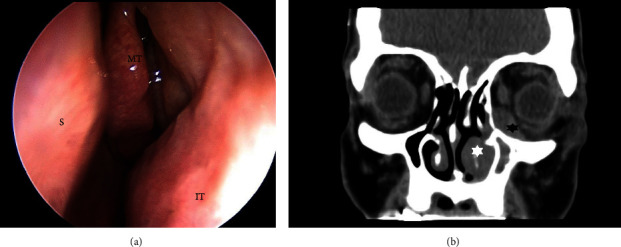
(a) Endoscopic image of the left nasal cavity that revealed the presence of pathologic tissue arising from the inferior turbinate and extending in the lateral nasal wall. (S: septum, MT: middle turbinate, and IT: inferior turbinate). (b) CT scan coronal view showing the presence of hypertrophic tissue of the lateral nasal wall and inferior turbinate (white star) extending inside the orbit (black star).

**Figure 2:**
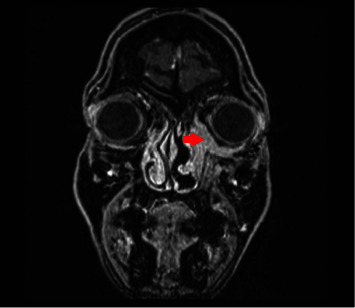
MRI coronal view revealing a lobulated mass with an anteroposterior diameter of 42 mm and a thickness of 13 mm located at the left lateral nasal wall and infiltrating the medial and lower border of the orbit without evident muscle infiltration (red arrow).

A 52-year-old woman presented to our clinic with a two-month history of dacryorrhea, mild nasal obstruction, periorbital pain, and slowly progressive periorbital edema. There was no history of epistaxis and no signs of ophthalmoplegia. Also, there was no history of fever nor mucopurulent discharge. The nasal endoscopy that was performed showed the presence of hyperplastic tissue arising from the inferior turbinate and the lateral nasal wall (Figure 1(a)). The CT scan revealed the presence of hyperplastic tissue at the level of the inferior left turbinate, the extension of this lesion through the lateral nasal wall inside the medial wall of the orbit, and mucosal thickening of the left maxillary sinus and anterior ethmoidal cells with inflammatory changes (Figure 1(b)). We started intravenous antibiotics and corticosteroids and prepared the patient for operation. We chose to perform the operation under general anesthesia because according to the findings of the preoperative CT a maxillary antrostomy and an anterior ethmoidectomy were scheduled. In the operating room, an endoscopic incisional biopsy resecting part of the inferior turbinate was performed. Also, we conducted a maxillary antrostomy to take biopsies from the maxillary sinus and an anterior ethmoidectomy. There were not any signs of pus presence inside the ethmoidal cells. The histopathological examination indicated the presence of seromucinous hamartoma in all the specimens. Postoperative MRI was executed (Figure 2) revealing a lobulated mass with an anteroposterior diameter of 42 mm and a thickness of 13 mm located at the left lateral nasal wall and infiltrating the medial and lower borders of the orbit without evident muscle infiltration. The patient was scheduled for surgery.

**Figure 3:**
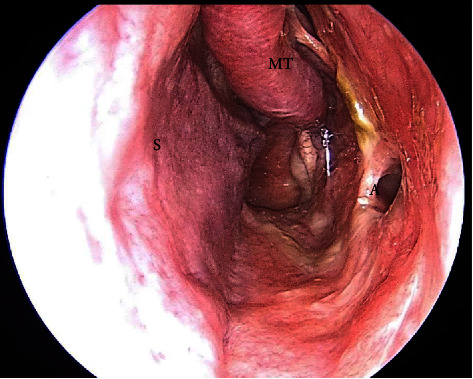
Endoscopic view with a 30° rigid endoscope of the left nasal cavity 16 months after the surgery showing no recurrence. (S: septum, A: antrostomy, and MT: middle turbinate).

Endoscopic medial maxillextomy with transnasal resection of the orbital lesion, which was in vicinity but did not invade the medial and inferior rectus, was performed. At the anterior border of resection, a cut was made from the anterior attachment of the middle turbinate to the lateral nasal wall downward to include the maxillary crest containing the nasolacrimal canal and duct. A mucosal flap was elevated with a Freer elevator, and an anterior osteotomy was made anteriorly to the nasolacrimal canal. Following osteotomy, the nasolacrimal duct, as it descends from the lacrimal sac, was sharply dissected with endoscopic scissors and included in the specimen. The lateral nasal wall was mobilized medially with progressive dissection allowing entry into the maxillary sinus. At this point, the infraorbital nerve canal was recognized and preserved. Posterior cuts were made posteriorly to the maxillary ostium at its junction with the orbit. The sphenopalatine artery was cauterized. The posterior attachment of the remaining part of the inferior turbinate was cut, and the specimen including the lateral wall with the tumor was removed. In the following surgical steps, the lamina papyracea and the adjacent periorbita were removed. The pathological tissue inside the orbit was removed, preserving the medial and inferior rectus muscles. The result of the biopsy from all the specimens was seromucinous hamartoma with no signs of malignancy. There were no postoperative complications, and the patient was discharged after 3 days with no signs of edema or pain. Sixteen months after the surgery during her regular follow up the patient is symptom-free with no signs of recurrence (Figure 3).

## 3. Discussion

The sinonasal tract encompasses a wide range of reactive and neoplastic lesions, and their differential diagnosis can be challenging, especially in small biopsy samples [12]. Different hamartomas of epithelial, mesenchymal, or mixed origin may arise in the sinonasal tract. They can be entirely of epithelial origin such as respiratory epithelial adenomatoid hamartoma (REAH), seromucinous hamartoma (SH), and the recently described olfactory epithelial hamartoma (OEH), of mixed epithelial and mesenchymal origin such as chondroosseous hamartoma and respiratory epithelial hamartoma (CORE), or of complete mesenchymal origin such as nasal chondromesenchymal hamartoma (NH) [3, 8, 12].

SH is considered a rare lesion of the sinonasal tract [5]. To date, about 45 cases of SH have been described in the literature through small case series and case reports (Table 1). It usually presents as a polypoid mass with a wide age range from 14 to 85 years with a peak in the 6^th^ decade of life. Although factors such as neoplastic proliferation, chronic mechanical irritation, inflammation, and embryological tissue misplacement are considered as possible mechanisms of its development, the exact pathogenesis remains still unclear [8]. In 80% of the cases, it is located in the posterior nasal septum and nasopharynx while the lateral nasal wall and sinuses are less common locations [10, 13]. It has a benign nature and is not usually associated with local invasion. Figures et al. demonstrated a case of SH arising from the nasal septum and involving the skull base exhibiting bone thinning [14]. In our patient, the lesion was located in the lateral nasal wall involving the inferior turbinate and the nasolacrimal duct, the maxillary sinus, and extending inside the orbit without invasion of the extraocular muscles. The most common symptoms are unilateral nasal obstruction, although bilateral location has been described in the literature [15], mucopurulent secretion, and epistaxis. However, in most cases, the patients are asymptomatic [11]. In our case, the patient presented with dacryorrhea, periorbital pain, and edema due to invasion of the nasolacrimal duct and the orbit. Nasal obstruction was not included in the main symptoms referred to by the patient, probably because the lesion did not extend so much inside the nasal cavity.

Pathological findings of SH, include an epithelial proliferation of small glands, serous acini, and tubules that can grow in clusters, lobules, or haphazardly. Larger glands or cysts covered by ciliated respiratory epithelium are frequently admixed with these small glands. The surrounding stroma is usually fibrous and often contains a lymphoplasmacytic inflammatory infiltrate lacking eosinophils. Periglandular hyalinisation and the absence of the myoepithelial layer have also been reported in some cases [3, 8–10]. The immunohistochemical staining of SH is positive for S-100, CK7, and CK19 but negative for CK14 and negative or focally positive for p63 and actin [3, 10].

The differential diagnosis includes REAH, low-grade sinonasal adenocarcinoma (LGSNA), juvenile nasopharyngeal angiofibroma (JNA), olfactory neuroblastoma (ON), and inflammatory nasal polyps [8, 10, 16]. JNA, unlike SH, usually arises from the sphenopalatine foramen and extends in the pterygopalatine fossa. Olfactory neuroblastoma is a malignant lesion originating from the superior part of the nasal cavity in the olfactory region which can be easily distinguished from SH with microscopical examination. Inflammatory polyps have a more edematous stroma and a smoother surface. In addition, the presence of numerous seromucinous glands distinguishes SH from inflammatory polyps of the nasal cavity [16]. REAH is the most common hamartoma of the sinonasal tract and was first described by Wenig and Heffer in 1995 [17]. It is a benign glandular proliferation with a polypoid appearance similar to that of inflammatory polyps but with a more rubbery surface. It is usually located in the olfactory cleft and posterior nasal septum, and it can appear as an isolated lesion or in association with other pathologies, including sinonasal polyposis, inverted papilloma, or malignancy [10]. Microscopic characteristics include medium-to-large glands connected to the surface, a frequently thickened basement membrane, and a multilayered ciliated respiratory epithelium, in contrast to SH, which has a single-layered epithelium [3, 10]. Immunohistochemistry reveals a ciliated epithelium positive for CK7 and basal cells positive for p63 and high-molecular-weight keratins.

Differential diagnosis from low-grade sinonasal adenocarcinoma (LGSNA) is important because it can impact therapeutical choice in terms of surgery extent and adjuvant radiotherapy. LGSNA exhibits an infiltrative exophytic pattern which usually grows in a papillary or cribriform shape that can invade nearby normal structures. Microscopically, it is characterized by the configuration of back-to-back glands with pleomorphisms, mitoses, anisocytosis, and cell hyperchromasia, without a rich stroma [3, 12]. SH is a benign neoplasm, and malignant transformation has not been reported yet [18–21]. However, Rengifo et al. displayed the case of a 39-year-old man with a left nasal mass which revealed to be a SH with focal areas of transition to low-grade adenocarcinoma characterized by stromal invasion but without bony, perineural, or lymphovascular invasion [22]. In our patient, the lesion extended inside the orbit probably through the nasolacrimal system, but there was no invasion of the muscles nor of the infraorbital nerve because the patient had no diplopia nor numbness or pain in the cheek. Part of the nasolacrimal duct was sharply dissected and included in the surgical specimen. We did not perform a concurrent endoscopic dacryocystorhinostomy (DCR), and until her most recent follow-up, the patient had no signs of epiphora. According to Sadeghi and Joshi, it seems that patients do not develop epiphora after endoscopic medial maxillectomy whether or not a DCR is performed [23].

The treatment of choice for this rare neoplasm is transnasal endoscopic resection, and with complete excision, recurrence is extremely rare [14].

## 4. Conclusion

Seromucinous hamartoma is a rare benign lesion of the sinonasal tract that should be included in the differential diagnosis of polypoid nasal lesions. Location in the lateral nasal wall is rare, to our knowledge, after a literature review, this is the first case with orbital involvement. Excision via transnasal endoscopic surgery is the treatment of choice today, and it can be combined with a transnasal approach of the orbit. Otorhinolaryngologists should be aware of this pathologic entity when dealing with nasal neoplasms.

## Figures and Tables

**Table 1:** Summary of the described cases with SH in the literature.

Author	Year	Number of patients	Location
Baillei et al.	1974	1	Septum
Zarbo et al.	1983	1	Nasopharynx
Graeme-Cook et al.	1992	3	Nasopharynx/Nasal cavity
Chuang et al.	2000	1	Sinus
Weinreb et al.	2009	7	Nasal cavity/septum
Ambrosini-Spaltro et al.	2010	5	Nasal cavity/septum
Figures et al.	2010	1	Nasal cavity
Khan et al.	2011	2	Nasal cavity
Fleming et al.	2012	1	Nasal cavity
Huang et al.	2015	2	Nasal cavity/septum
Huang et al.	2017	7	Nasal cavity/septum
Chen Bo-Nien et al.	2017	1	Nasal cavity bilateral
Lee DH et al.	2018	1	Nasal cavity (inferior turbinate)
Tong et al.	2019	1	Nasal cavity
Sahin et al.	2020	1	Nasal cavity
Baneckova et al.	2020	5	Nasal cavity
Peric et al.	2020	1	Nasal cavity (middle turbinate)
Pauna et al.	2020	1	Nasal cavity
Alokby et al.	2021	1	Sinus
Godse et al.	2021	1	Sinus
Rengifo et al.	2021	1	Nasal cavity/sinus
Total		45	

## Data Availability

The data used to support the findings of this study are included within the article.
